# PReS-FINAL-2175: Pharmacokinetics of mycophenolate mofetil in children with lupus and clinical findings in favour of therapeutic drug monitoring

**DOI:** 10.1186/1546-0096-11-S2-O10

**Published:** 2013-12-05

**Authors:** JB Woillard, B Bader-Meunier, R Salomon, B Ranchin, S Decramer, M Fischbach, E Berard, F Saint-Marcoux

**Affiliations:** 1 CHU Limoges, Limoges, France; 2 Hôpital Necker, Paris, France; 3 Hôpital Femme-Mère-Enfants, Lyon, France; 4 Hôpital Purpan, Toulouse, France; 5 Hôpital Hautepierre, Strasbourg, France; 6 Hôpital de Lenval, Nice, France; 7 Hôpital de Limoges, Limoges, France

## Introduction

The use of mycophenolate mofetil (MMF) in children with Systemic Lupus Erythematosus (SLE) is increasing. However, the clinical benefit of its monitoring has been scarcely studied, and little is known about its pharmacokinetics (PK) in this context.

## Objectives

To describe mycophenolic acid (MPA) PK, and to explore the relationships between exposure indices to MPA and the clinical status in children with SL

## Methods

This retrospective study included 36 children with SLE already treated with a maintenance immunosuppressive therapy including MMF. Full-PK profiles of MPA were modelled using an iterative two-stage approach and a simulation approach was applied for model refitting and validation. Using this a priori PK information, a Bayesian estimator was then developed that could allow accurate determination of MPA PK and exposure using 3 blood samples. The relationships between MPA through concentrations (C0), Area under the Curve (AUC) or AUC/dose values, and the activity of the disease (expressed using the consensually recommended Systemic Lupus Erythematosus Disease Activity Index; SLEDAI) were explored using Receiver Operating Curve (ROC) and logistic regression analysis.

## Results

the ROC curve analysis showed that AUC ≤ 44 mg*h/L and AUC/dose< = 0.06 mg*h/L/mg were associated with the best diagnostic performance (Sensitivity of 78% and 94% and specificity of 94% and 56% respectively). When introduced in logistic regression model these threshold were associated with an increased risk of active disease (AUC/dose < 0.06, OR[95%CI] = 59.5 [5.9-588.2], p = 0.0005; AUC < 44 mg.h/L, OR = 21.2 [2.3-196.1].

## Conclusion

AUC> 45 mg * h/L or AUC/dose> 0.06 could be proposed as a target AUC to limit relapse of the disease or its progression in further clinical trials.

## Disclosure of interest

None declared.

